# Eco-friendly nitrogen-containing carbon encapsulated LiMn_2_O_4_ cathodes to enhance the electrochemical properties in rechargeable Li-ion batteries

**DOI:** 10.1038/srep29826

**Published:** 2016-07-13

**Authors:** P. Robert Ilango, K. Prasanna, Su Jung Do, Yong Nam Jo, Chang Woo Lee

**Affiliations:** 1 Department of Chemical Engineering, College of Engineering, Kyung Hee University, 1732 Deogyeong-daero, Gihung, Yongin, Gyeonggi 17104, South Korea

## Abstract

This study describes the synthesis of nitrogen-containing carbon (N-C) and an approach to apply the N-C material as a surface encapsulant of LiMn_2_O_4_ (LMO) cathode material. The N heteroatoms in the N-C material improve the electrochemical performance of LMO. A low-cost wet coating method was used to prepare N-C@LMO particles. The N-C@LMO was characterized by X-ray diffraction (XRD), X-ray photoelectron spectroscopy (XPS), thermogravimetric analysis (TGA), high-resolution Raman spectroscopy (HR-Raman), field emission scanning electron microscopy (FE-SEM), and field emission scanning transmission electron microscopy (FE-TEM) with elemental mapping. Furthermore, the prepared samples were electrochemically studied using the AC electrochemical impedance spectroscopy (EIS) and the electrochemical cycler. XPS suggested that the N-C coating greatly reduced the dissolution of Mn and EIS showed that the coating greatly suppressed the charge transfer resistance, even after long-term cycling. The control of Mn dissolution and inner resistance allowed faster Li-ion transport between the two electrodes resulting in improved discharge capacity and cycling stability.

To meet the inevitable energy requirement of electronic goods, electric vehicles (EV), and hybrid electric vehicles (HEV), diverse studies have been carrying out to upgrade the current energy materials in rechargeable Li-ion technologies. Particularly, extensive research has been conducted to improve cathode active materials toward the goal of increasing energy storage, which would be applicable to mass-scale production of Li-ion batteries. Spinel LMO is one of the most promising candidates for the cathode material in rechargeable Li-ion batteries owing to its low cost, safety, and abundance^1^,^2^,^3^. Although LMO is promising for sourcing power to electronic appliances, there are some problems with its use, including dissolution of Mn through a disproportionation reaction (2Mn^3+^→ Mn^2+^ + Mn^4+^), phase transition from cubic to tetragonal, and formation of oxygen vacancies due to Mn dissolution^4^,^5^,^6^. Various strategies to address these problems have been proposed, including synthesis of various morphologies^7^, doping^8^, and coating^9^. Among them, coating is a facile way to improve LMO’s innate properties^10^,^11^,^12^. Specifically, carbon coating has received the most attention because the carbon itself can provide a continuous electron pathway through the coating, allowing the particles to remain electrically conductive^13^. Moreover, coating of carbon or carbon-like materials on LMO has actually enhanced its electrochemical properties. For example, *Han, A. R. et al*. reported that LMO coated with a sucrose-derived carbon coating was electrochemically more stable than uncoated material^14^. *Zhuo, H. et al*. carried out coating with a graphene-like membrane (liquid polyacrylonitrile) as a surface-modifying agent and demonstrated that this coating considerably improved discharge capacity and cycling stability^15^. *Tang, M. et al*. applied carbon nanotubes (CNTs) as a coating material and demonstrated their high-power capability^16^ and *Bak, S.-M. et al*. studied reduced graphene oxide (RGO) as a coating and showed that it enabled high surface charge storage^17^. *Ju, B. et al*. used graphene supported with Y_2_O_3_ as an alternative surface material, and noted its suppression of Mn^3+^ dissolution and resulting good retention characteristics^18^. Although these previously reported coating approaches yield better electrochemical performance than uncoated materials, the selected coating material and the synthesis process seem to be expensive and complicated. On the other hand, materials of carbon doped with heteroatoms such as iodine, boron, and nitrogen have been introduced as anode materials for use in rechargeable Li-ion batteries, and have been reported to yield high reversible capacity, stability under prolonged cycling, and rapid surface Li-ion adsorption^19^,^20^. It is well known that both CNTs and RGO are expensive to synthesize, requiring long physical and chemical procedures. When alternative materials exist that are abundant, readily available, and eco-friendly, and that yield results comparable to those of high-cost materials, such materials will always be valued; such alternatives to CNTs and RGO have been reported for various applications including sensors^21^,^22^, supercapacitors^23^, and Li-ion batteries^24^. Eco-friendly materials are considered to be those that reduce or control the usage of hazardous materials. Many conductive polymer materials have been used as carbon sources, such as polyparaphenylene^25^, polyaniline^26^, and polydioxyethylenethiophene^27^ in Li-ion batteries. Natural polymer materials such as chitin and chitosan may also be applied. Chitosan is an interesting polysaccharide because of its distinct amino and hydroxyl groups^28^,^29^. Recently, N-C matrix material prepared from chitosan has been identified as an electrically conductive material and has been applied to Li-ion batteries and supercapacitors, yielding excellent electrochemical characteristics^30^,^31^,^32^. However, there have been no reports on chitosan-derived N-C@LMO for use as a cathode material in Li-ion batteries. Accordingly, in the present work we modified LMO with N-C and investigated the effects of this surface encapsulation. Moreover, we systematically investigated the structure of bonding between LMO and N-C, as well as the contribution of N in the N-C material.

## Results

### Structure and morphology

**Figure 1:**
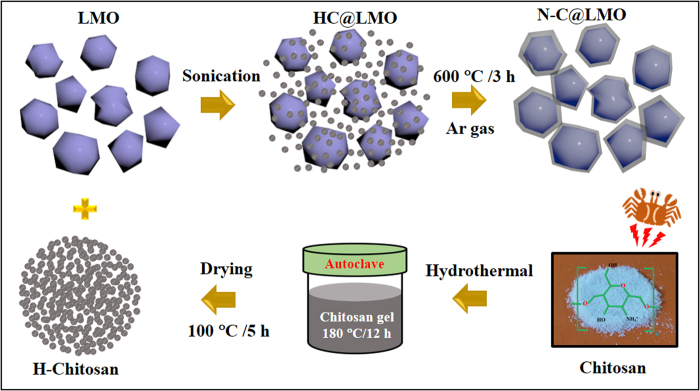
Schematic diagram for preparation of N-C@LMO sample.

The method used to prepare N-C@LMO is schematically illustrated in Fig. 1; it included four basic steps as follows. In the first step, chitosan was dissolved in a solution of acetic acid in deionized water. The second step was a hydrothermal reaction under fixed parameters, and the third step was ultrasonication treatment to yield a uniform dispersion. At this stage, the H-chitosan would be uniformly distributed over the LMO surface; as a matter of fact, the –NH_3_^+^ and –OH functional groups of H-chitosan easily bind with the LMO surface. Finally, calcination was carried out; this is the most vital step in this experiment because under high temperature the functional groups will become attached by strong physical interaction to the edges of the LMO particles, leading to the formation of N-C@LMO.

**Figure 2:**
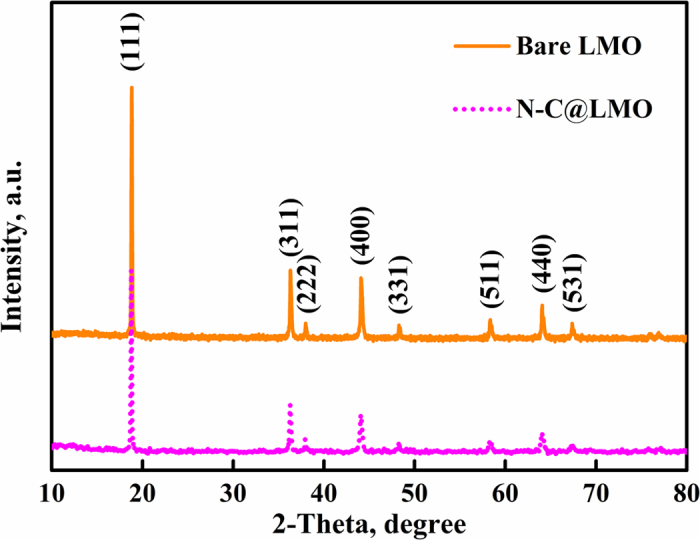
XRD patterns for bare LMO and N-C@LMO samples.

Phase evaluations of prepared bare LMO and N-C@LMO samples are shown in Fig. 2; sharp and strong peaks revealed that the bare LMO and N-C@LMO samples were finely crystalized in a pure cubic phase of the Fd3m space group (JCPDS card no. 89-0118). After coating, no peak from carbon could be observed in diffraction patterns, which could be attributed to its small quantity as well as its amorphous character^33^. However in the magnified view of 111 planes shown in Fig. S1 a negligible change is observed.

**Figure 3:**
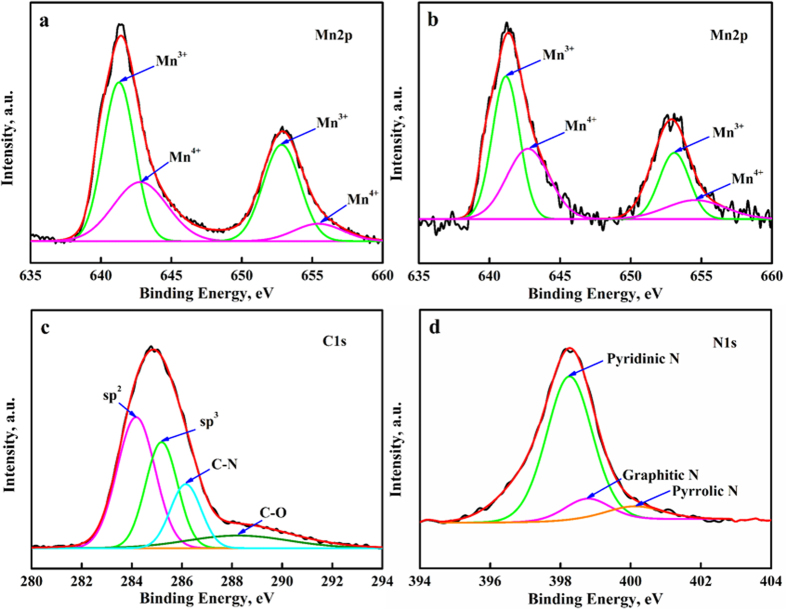
XPS spectra (**a,b**) high resolution of Mn2p for bare LMO and N-C@LMO samples, (**b,c**) C1s and N1s spectra for N-C@LMO sample.

The surface compositions of prepared samples were analyzed by XPS, as shown in Fig. 3. XPS is an effective tool to characterize elements on the surface, yielding specific information on binding energy and oxidation states^34^. XPS survey scan spectra of both bare LMO and N-C@LMO samples are given in Fig. S2; these spectra confirmed the presence of Mn, O, C, and N. Due to spin–orbit splitting, the Mn2p spectral peaks were clearly separated into the two oxidation states of Mn2p_3/2_ and Mn2p_1/2_, by about 11.5 eV. These splitting binding energies agree quite closely with those of a previous report^35^. Figure 3a shows the deconvoluted values by fitting for bare LMO sample; the binding energies of Mn^3+^ were 641.29 eV (Mn2p_3/2_) and 652.82 eV (Mn2p_1/2_), whereas those of Mn^4+^ were 642.86 (Mn2p_3/2_) and 655.42 (Mn2p_1/2_). Figure 3b shows the corresponding spectra for the N-C@LMO sample; the binding energies of Mn^3+^ were 641.18 (Mn2p_3/2_) and 653.11 (Mn2p_1/2_), and those of Mn^4+^ were 642.72 (Mn2p_3/2_) and 654.54 (Mn2p_1/2_). Variation in binding energies are due to the N-C coating^36^. The ratio of Mn^3+^/Mn^4+^ was lower for N-C@LMO than for bare LMO. Generally, the electrochemically active Mn^3+^ is not stable, but after coating it was stable; this was attributed to suppression of Mn^3+^ dissolution by the coating. The deconvoluted C1s peaks demonstrated in Fig. 3c. The peaks located at 284.1 eV, 285.2 eV, 286.1 eV and 288.3 eV corresponding to sp^2^, sp^3^ hybridized carbon, C-N and C=O, respectively^37^. Figure 3d gives a high-resolution view of the N1s peak, which comprised contributions by pyridinic and pyrrolic N^38^; the binding energies of 398.2 eV, 398.7 eV, and 400.2 eV respectively correspond to pyridinic, graphitic, and pyrrolic N. The presence of such N groups at the graphite edges can generate vacancies in the carbon matrix; these vacancies will increase the diffusion speed of Li-ions^13^.

**Figure 4:**
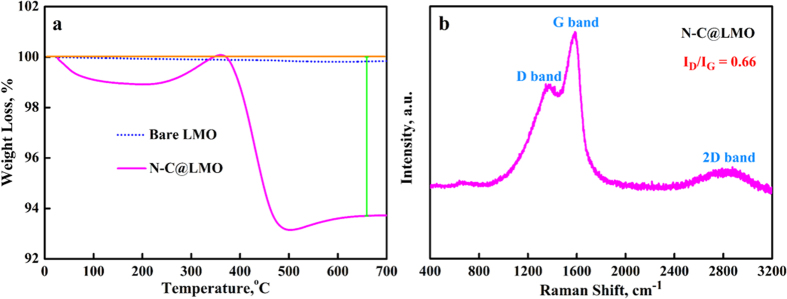
The profile (**a**) TGA for bare LMO and N-C@LMO samples measured in air atmosphere with a heating rate of 10 °C/min and (**b**) HR-Raman spectra of N-C@LMO.

Figure 4a displays the TGA profile collected for the N-C@LMO sample, which obviously shows weight loss of carbon materials during TGA in air. The calculated mass of carbon on the LMO was ~6.3 wt%. Figure 4b shows the HR-Raman spectrum of the N-C@LMO sample, which included a D band at ~1368 cm^−1^, a G band at ~1582 cm^−1^, and a peak over the range from ~2590 to ~3060 cm^−1^ corresponding to the 2D band; these are typical peaks for graphene-like carbon. The intensity ratio I_D_/I_G_, which indicates the degree of disorder of the carbon, was ~0.66. The bands D, G, and 2D respectively arise from first-order zone boundary phonons, in-plane optical vibrations and second-order zone boundary phonons. The loss of long-range ordering between graphene layers gives combined D and G bands^15^,^19^,^39^. Peaks in the range of 500–700 cm^−1^ indicate metal–oxygen stretching vibrations^40^. In addition, the Raman spectrum of the H-chitosan is shown in Fig. S3. It has similar behavior of N-C@LMO and I_D_/I_G_ value was ~0.65.

**Figure 5:**
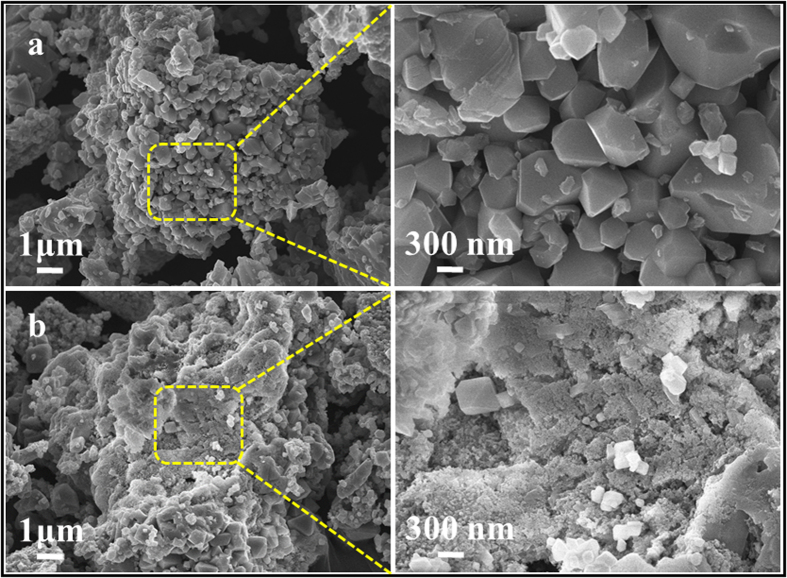
FE-SEM images (**a**) bare LMO and (**b**) N-C@LMO samples with different magnifications.

The surface morphologies of prepared samples were investigated by means of FE-SEM; Fig. 5 shows FE-SEM images of bare LMO and N-C@LMO samples. The surface of the bare LMO sample seemed to be smooth, with no surface diversion on individual primary LMO particles, and showed well-defined crystallization. Contrastingly, the N-C@LMO sample was not smooth, exhibiting layered materials as an indication of the surface encapsulation.

**Figure 6:**
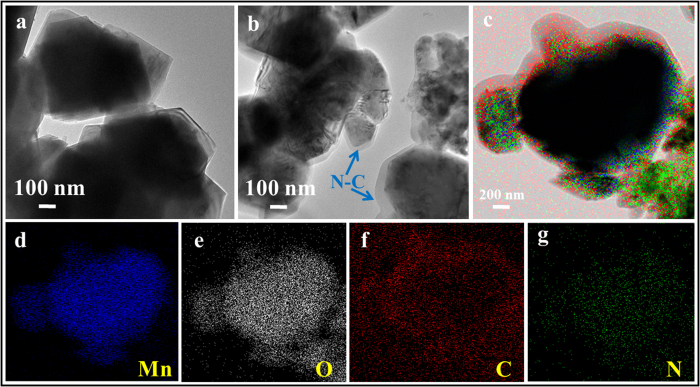
FE-TEM images (**a**) bare LMO, (**b**) N-C@LMO, and (**c–g**) mapping analysis for Mn, O, C, and N.

To improve our understanding of phenomena related to the N-C@LMO, we conducted the further analyses of FE-TEM and elemental mapping Fig. 6. The N-C@LMO sample edges appeared quite different from bare LMO due to the thin decoration, as indicated with arrows in the Fig. 6b. The amorphous N-C surface layer was ~20–25 nm thick. Figure 6c shows an overlay of EDS mapping on the N-C@LMO surface. This mapping elucidated the presence of Mn, O, C, and N, which were uniformly distributed in the magnified area shown (Fig. 6(d–g)). This kind of N-C matrix improves the electrochemical properties of carbonaceous material^41^ and showed good overlapping with all the elements throughout the entire material.

### Electrochemical analysis

**Figure 7:**
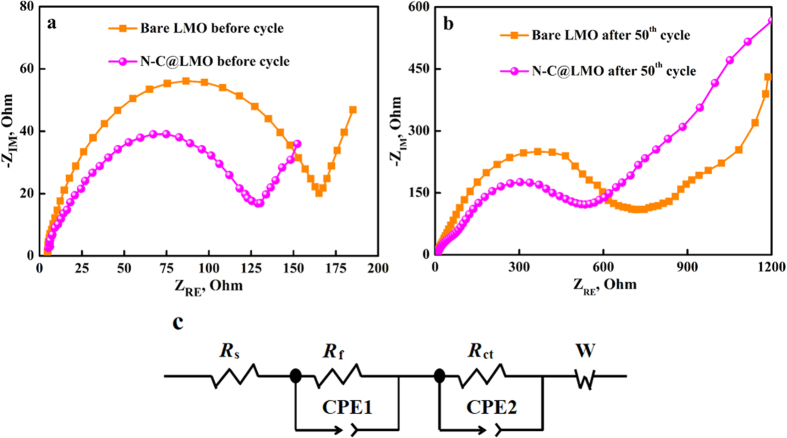
EIS response (**a**) before cycle (**b**) after 50^th^ cycle, and (**c**) An equivalent circuit to fit the impedance signal.

EIS measurements were performed to evaluate the impedance behavior of the N-C@LMO sample. Figure 7a,b show the impedance signals collected for bare LMO and N-C@LMO before and after cycling. An equivalent circuit model was used to fit the impedance signal (Fig. 7c). This circuit included *R*_s_, the ohmic resistance of the electrolyte; *R*_ct_, the charge transfer resistance; CPE, the double layer capacitance and passivation film capacitance; and *Z*_w_, the Warburg impedance^42^,^43^,^44^. Before cycling, the *R*_ct_ values for bare LMO and N-C@LMO were 165 Ω and 130 Ω, respectively. To study the effect of the coating, we characterized cells after fully charging them to 4.5 V after 50 cycles; the *R*_ct_ values for bare LMO and N-C@LMO were then 536 Ω and 730 Ω. *R*_ct_ increased during the cycling due to the formation of an interfacial layer between the electrode and the electrolyte. From the Nyquist plot observations it is understood that the N-C@LMO sample has improved Li-ion conduction compared with the bare LMO. Faster Li-ion diffusion directly results in enhanced electronic conductivity. This phenomenon could be explained as follows. (і) N atoms, which each have an excess valence electron, may donate additional π electrons to graphitic planes; (іі) the difference in electronegativity between N and C reduces the work function, and (ііі) N doping produces surface capacitive effects. Thus, we confirmed through AC impedance analysis that the N-C precursors yield better electronic conductivity than the pure carbonaceous materials^45^,^46^,^47^.

**Figure 8:**
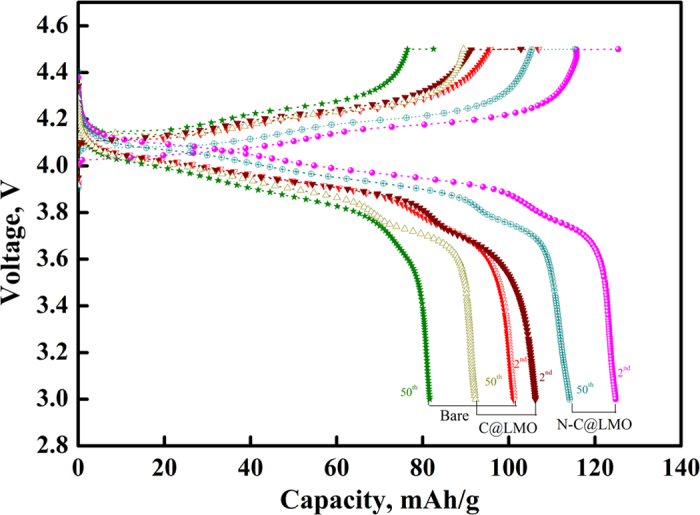
The galvanostatic potential profiles for bare LMO, C@LMO, and N-C@LMO samples between the voltage limits of 3.0 V and 4.5 V at 1 C rate.

Galvanostatic potential curves for bare LMO, C@LMO, and N-C@LMO samples were collected over the voltage range of 3.0 to 4.5 V vs. Li/Li^+^ and at the rate of 1C; the results are shown in Fig. 8. These curves had two noticeable plateaus, in agreement with a two-phase model^48^.


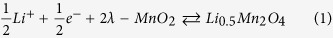



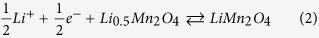


In detail, during the charge process, half of the Li^+^ions de-intercalated from the tetrahedral sites in LMO through Li–Li interactions, leading to the formation of a Li_0.5_Mn_2_O_4_ intermediate; this reaction is associated with the first charge plateau^49^. Then, further de-intercalation of Li from the tetrahedral sites happened without the aid of any Li-Li interaction, giving rise to a product with the final composition of λ-MnO_2_; this can be associated with the second charge plateau. Both curves were analyzed in detail, and it was found that the potential plateau of bare LMO showed strong polarization after the second and 50th cycles, when compared with C@LMO and N-C@LMO. In specific, the N-C@LMO sample’s polarization effect seemed to be shifted in the negative direction after the second cycle, and even after many cycles it was still lower than that of the bare LMO and C@LMO. The lower potential difference between charge and discharge plateau denotes better Li-ion conduction, suppressed polarization increment and inner resistance in the cell. Particularly, coating played a main role in (і) isolation of the electrode from the electrolyte, (іі) suppressing the outbreak of the salts LiF and Li_2_CO_3_ from the electrolyte, (ііі) regulating the Mn dissolution in the electrolyte, and (iv) improving the conductivity^44^,^50^,^51^. In addition to the carbon coating the presence of N facilitated fast Li^+^ intercalation and de-intercalation during the electrochemical reactions. The carbon encapsulation provides catalytic action by decreasing the local concentration of HF acid^12^. The formation of HF as a byproduct proceeds by the following reaction^52^.


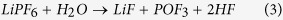


The HF thus formed will attack the active material as follows.


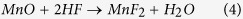


This kind of attack will create a layer on the surface of the cathode active material, eventually leading to electrolyte decomposition. This mechanism causes severe capacity fading during electrochemical cycling as follows^53^,^54^


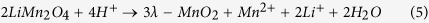


**Figure 9:**
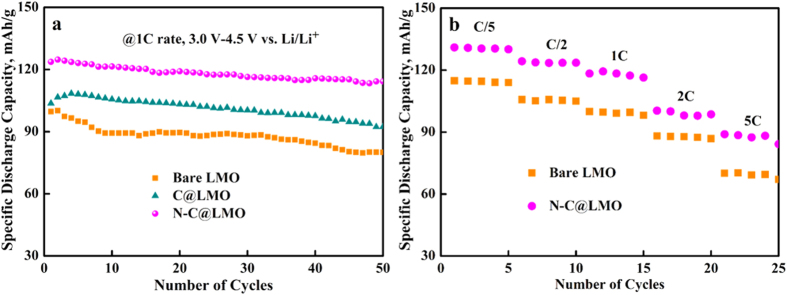
The profile: (**a**) Cycling performance for bare LMO, C@LMO and N-C@LMO and (**b**) rate capability for bare LMO, and N-C@LMO samples between the voltage limits of 3.0 V and 4.5 V.

Figure 9 shows the cycling performance of bare LMO, C@LMO, and N-C@LMO samples cycled at the rate of 1C over the voltage range of 3.0 to 4.5 V vs. Li/Li^+^. It can be clearly seen that the N-C@LMO sample delivered the higher discharge capacity of ~123.6 mAh/g during the first cycle and with capacity retention of ~92% over 50 cycles, comparable to or greater than that of previous reports^55^,^56^. Contrastingly, bare LMO yielded the relatively poor discharge capacity of ~100.4 mAh/g for the first cycle and also poor capacity retention of ~80% over 50 cycles. This may impact the intrinsic properties of LMO in high voltage ranges. Evidently, N-C@LMO yields excellent cycling stability with improved discharge capacity because the use of a C matrix including N improved the electron diffusion. This electron pathway facilitated the adsorption of Li^+^ on the surface of the LMO, resulting in improved electrochemical properties. To specify, the impact of N in the carbon, the cycling behavior of N-C@LMO has been compared with C@LMO. The sample C@LMO delivers the discharge capacity of ~103.6 mAh/g during the first cycle with the capacity retention of 89% over 50 cycles, which is comparatively lower than the N-C@LMO samples. A high rate capability is always a key criterion to assess the performance of cathode materials used in high-power, high-energy Li-ion batteries. The rate capability of bare LMO and N-C@LMO samples were demonstrated in Fig. 9(b). All the rate tests were examined between the voltage range of 3.0 and 4.5 V vs. Li/Li^+^. The rate performances of N-C@LMO sample shows quite higher discharge capacity than the bare LMO. This results giving proof to improvement of structure stability and faster Li^+^ diffusion especially at high current rates. The surface-encapsulated LMO cathodes experience less loss of the initial capacity, and better cycling performance.

## Discussion

We successfully synthesized N-C by means of a hydrothermal method, and used this material as a surface encapsulant of LMO cathodes, using a simple wet coating treatment. The N-C coating meaningfully enhanced the discharge capacity and cyclability of the LMO cathodes. N-C material was easily coated onto the LMO and produced an orderly surface encapsulation that promoted fast Li-ion diffusion by maintaining the LMO core material’s structure. The coating suppressed Mn dissolution and prevented direct contact between the electrode and electrolyte. Observed EIS and cycling behaviors showed the promise of this coating method to yield a high-performance cathode material for Li-ion rechargeable batteries. Moreover, the novel ecofriendly biopolymer used herein will be good choice in designing various energy storage materials for future automotive applications.

## Experimental

### Synthesis of N-C material

The N-C material was synthesized by means of a facile hydrothermal method. Briefly, 2 g of chitosan (obtained from Sigma-Aldrich Co., Ltd.) was dissolved in deionized water, followed by addition of 0.5 ml of acetic acid (CH_3_COOH). This mixture was stirred for a long time to allow the chitosan to completely dissolve. The resulting polymeric gel was transferred into a Teflon-lined tube (100 ml capacity), which was then placed in a stainless steel autoclave, heated to 180 °C, and held at this temperature for 12 h. The reaction mixture was allowed to cool to room temperature and the resulting black particles were collected and then washed once each with distilled water and ethanol. This hydrothermally treated chitosan (hereafter termed H-chitosan) was dried at 100 °C for 5 h prior to use as a coating agent.

### Preparation of N-C@LMO and C@LMO

Surface encapsulation of LMO by N-C material was carried out using a simple wet coating method. First, LMO (obtained from Ecopro Co., Ltd.) was dispersed in deionized water with the help of ultrasonication for 0.5 h. Then, an appropriate amount of as-prepared H-chitosan was added to the dispersion and ultrasonicated for about 1 h. Then, the solvent was removed by evaporation at 70 °C under stirring. Finally, the samples were collected and calcinated at 600 °C for 3 h under Ar atmosphere. For comparison, same experiment has been conducted with glucose as carbon source. Hereafter, the uncoated, carbon coated and nitrogen contained carbon coated LMO materials are respectively termed bare LMO, C@LMO and N-C@LMO.

### Characterization

The attempted materials were characterized by means of various analysis as follows. XRD (D8Discover with GADDS, Bruker AXS) was carried out over the 2θ range of 10–80°, using a CuKα radiation source (λ = 1.5406 Å). XPS analysis was carried out using a K-Alpha model (Thermo Electron). Other characterization techniques included FE-SEM (Leo Supra 55, Genesis 2000, Carl Zeiss), FE-TEM JEM-2100F, JEOL) with elemental mapping, TGA (DTG-60H thermal analyzer, Shimadzu), high-resolution Raman spectroscopy (HR-Raman, inVia), EIS (IVIUM), and electrochemical (Arbin).

### Cell fabrication

Cells of CR2032 standard form were assembled to analyze the electrochemical behavior of each sample. Electrodes were prepared by blending 80 wt% N-C@LMO (active material), 10 wt% carbon black (conductive agent), and 10 wt% polyvinylidene fluoride (binder) in N-methyl-2-pyrrolidone (NMP) solvent to form a slurry. The mixture was uniformly laminated onto Al foil and dried for 12 h at 120 °C in a vacuum oven. This electrode was cut to form a small disk shape of the required diameter. The electrochemical cells were assembled in a high-purity argon atmosphere (<1 mg/L of O_2_ and H_2_O). Each cell comprised a cathode prepared as described above, a Li metal anode, electrolyte (1 M LiPF_6_ in a solvent of 1:1 EC:DEC by volume), and a Celgard 3501 separator.

## Additional Information

**How to cite this article**: Ilango, P. R. *et al*. Eco-friendly nitrogen-containing carbon encapsulated LiMn_2_O_4_ cathodes to enhance the electrochemical properties in rechargeable Li-ion batteries. *Sci. Rep.* **6**, 29826; doi: 10.1038/srep29826 (2016).

## Supplementary Material

Supplementary Information

## References

[b1] Cheng F. . Porous LiMn_2_O_4_ nanorods with durable high-rate capability for rechargeable Li-ion batteries. Energy Environ. Sci. 4, 3668–3675 (2011).

[b2] Scrosati B. & Garche J. Lithium batteries: Status, prospects and future. J. Power Sources 195, 2419–2430 (2010).

[b3] Manthiram A., Vadivel Murugan A., Sarkar A. & Muraliganth T. Nanostructured electrode materials for electrochemical energy storage and conversion. Energy Environ. Sci. 1, 621–638 (2008).

[b4] Ding Y.-L. . Single-Crystalline LiMn_2_O_4_ Nanotubes Synthesized Via Template-Engaged Reaction as Cathodes for High-Power Lithium Ion Batteries. Adv. Funct. Mater. 21, 348–355 (2011).

[b5] Thackeray M. M. Spinel Electrodes for Lithium Batteries. J. Am. Ceram. Soc. 82, 3347–3354, (1999).

[b6] Xi L. J. . Facile synthesis of porous LiMn_2_O_4_ spheres as positive electrode for high-power lithium ion batteries. J. Power Sources 198, 251–257 (2012).

[b7] Bao S.-J., Li C.-M., Li H.-L. & Luong J. H. T. Morphology and electrochemistry of LiMn_2_O_4_ optimized by using different Mn-sources. J. Power Sources 164, 885–889 (2007).

[b8] Xiao L. . Enhanced electrochemical stability of Al-doped LiMn_2_O_4_ synthesized by a polymer-pyrolysis method. Electrochim. Acta 54, 545–550 (2008).

[b9] Chang M. H. & Lee C. W. Direct contact shield of LiMn_2_O_4_ active material from electrolyte. Surf. Rev. Lett 17, 81–86 (2010).

[b10] Zhao J. & Wang Y. Ultrathin Surface Coatings for Improved Electrochemical Performance of Lithium Ion Battery Electrodes at Elevated Temperature. J. Phys. Chem. C 116, 11867–11876 (2012).

[b11] Hung F.-Y., Lui T.-S. & Liao H.-C. A study of nano-sized surface coating on LiMn_2_O_4_ materials. Appl. Surf. Sci. 253, 7443–7448 (2007).

[b12] Lee C. W., Kim H.-S. & Moon S.-I. Effects on surface modification of spinel LiMn_2_O_4_ material for lithium-ion batteries. Mater. Sci. Eng B 123, 234–237 (2005).

[b13] Long D. H. . Coating Lithium Titanate with Nitrogen-Doped Carbon by Simple Refluxing for High-Power Lithium-Ion Batteries. ACS Appl. Mater. Interfaces 7, 10250–10257 (2015). 25923036 10.1021/acsami.5b00776

[b14] Han A. R., Kim T. W., Park D. H., Hwang S.-J. & Choy J.-H. Soft Chemical Dehydration Route to Carbon Coating of Metal Oxides: Its Application for Spinel Lithium Manganate. J. Phys. Chem. C 111, 11347–11352 (2007).

[b15] Zhuo H. . Improved electrochemical performance of spinel LiMn_2_O_4_ *in situ* coated with graphene-like membrane. J. Power Sources 247, 721–728 (2014).

[b16] Tang M., Yuan A., Zhao H. & Xu J. High-performance LiMn_2_O_4_ with enwrapped segmented carbon nanotubes as cathode material for energy storage. J. Power Sources 235, 5–13 (2013).

[b17] Bak S.-M. . Spinel LiMn_2_O_4_/reduced graphene oxide hybrid for high rate lithium ion batteries. J. Mater. Chem. 21, 17309–17315 (2011).

[b18] Ju B. . Electrochemical performance of the graphene/Y_2_O_3_/LiMn_2_O_4_ hybrid as cathode for lithium-ion battery. J. Alloy. Comp. 584, 454–460 (2014).

[b19] Zhan Y. . Iodine doped graphene as anode material for lithium ion battery. Carbon 94, 1–8 (2015).

[b20] Wu Z.-S., Ren W., Xu L., Li F. & Cheng H.-M. Doped Graphene Sheets As Anode Materials with Super high Rate and Large Capacity for Lithium Ion Batteries. ACS Nano 5, 5463–5471 (2011). 21696205 10.1021/nn2006249

[b21] Nichols S. P., Koh A., Storm W. L., Shin J. H. & Schoenfisch M. H. Biocompatible Materials for Continuous Glucose Monitoring Devices. Chem. Rev. 113, 2528–2549 (2013). 23387395 10.1021/cr300387j PMC3624030

[b22] Madhu R., Sankar K. V., Chen S.-M. & Selvan R. K. Eco-friendly synthesis of activated carbon from dead mango leaves for the ultrahigh sensitive detection of toxic heavy metal ions and energy storage applications. RSC Adv. 4, 1225–1233 (2014).

[b23] Senthilkumar S. T., Selvan R. K., Melo J. S. & Sanjeeviraja C. High Performance Solid-State Electric Double Layer Capacitor from Redox Mediated Gel Polymer Electrolyte and Renewable Tamarind Fruit Shell Derived Porous Carbon. ACS Appl. Mater. Interfaces 5, 10541–10550 (2013). 24164312 10.1021/am402162b

[b24] Jiang J. . Evolution of disposable bamboo chopsticks into uniform carbon fibers: a smart strategy to fabricate sustainable anodes for Li-ion batteries. Energy Environ. Sci. 7, 2670–2679, (2014).

[b25] Chen Y. . Li^+^-Conductive Polymer-Embedded Nano-Si Particles as Anode Material for Advanced Li-ion Batteries. ACS Appl. Mater. Interfaces 6, 3508–3512 (2014). 24467155 10.1021/am4056672

[b26] Chen W.-M., Huang Y.-H. & Yuan L.-X. Self-assembly LiFePO_4_/polyaniline composite cathode materials with inorganic acids as dopants for lithium-ion batteries. J. Electroanal. Chem. 660, 108–113 (2011).

[b27] Liu X., Li H., Li D., Ishida M. & Zhou H. PEDOT modified LiNi_1/3_Co_1/3_Mn_1/3_O_2_ with enhanced electrochemical performance for lithium ion batteries. J. Power Sources 243, 374–380 (2013).

[b28] Anastas P. & Eghbali N. Green Chemistry: Principles and Practice. Chem. Soc. Rev. 39, 301–312 (2010). 20023854 10.1039/b918763b

[b29] Pillai C. K. S., Paul W. & Sharma C. P. Chitin and chitosan polymers: Chemistry, solubility and fiber formation. Prog. Polym. Sci. 34, 641–678 (2009).

[b30] Prasanna K., Subburaj T., Jo Y. N., Lee W. J. & Lee C. W. Environment-Friendly Cathodes Using Biopolymer Chitosan with Enhanced Electrochemical Behavior for Use in Lithium Ion Batteries. ACS Appl. Mater. Interfaces 7, 7884–7890 (2015). 25822540 10.1021/am5084094

[b31] Yue L., Zhang L. & Zhong H. Carboxymethyl chitosan: A new water soluble binder for Si anode of Li-ion batteries. J. Power Sources 247, 327–331 (2014).

[b32] Deng X., Zhao B., Zhu L. & Shao Z. Molten salt synthesis of nitrogen-doped carbon with hierarchical pore structures for use as high-performance electrodes in supercapacitors. Carbon 93, 48–58 (2015).

[b33] Jiang Q., Wang X. & Tang Z. Improving the Electrochemical Performance of LiMn_2_O_4_ by Amorphous Carbon Coating. Fullerenes, Nanotubes and Carbon Nanostruct. 23, 676–679 (2015).

[b34] Ming H. . Gradient V_2_O_5_ surface-coated LiMn_2_O_4_ cathode towards enhanced performance in Li-ion battery applications. Electrochim. Acta 120, 390–397 (2014).

[b35] Moses Ezhil Raj A. . XRD and XPS characterization of mixed valence Mn_3_O_4_ hausmannite thin films prepared by chemical spray pyrolysis technique. Appl. Surf. Sci. 256, 2920–2926 (2010).

[b36] Wang H.-Q. . Excellent stability of spinel LiMn_2_O_4_-based cathode materials for lithium-ion batteries. Electrochim. Acta 177, 290–297 (2015).

[b37] Hao P. . Graphene-based nitrogen self-doped hierarchical porous carbon aerogels derived from chitosan for high performance supercapacitors. Nano Energy 15, 9–23 (2015).

[b38] Zhang J., Ni S., Ma J., Yang X. & Zhang L. High capacity and super long cycle life of Li_3_VO_4_/N–C hybrids as anode for high performance Li-ion batteries. J. Power Sources 301, 41–46 (2016).

[b39] Zhu J. . Nitrogen-doped carbon nanofibers derived from polyacrylonitrile for use as anode material in sodium-ion batteries. Carbon 94, 189–195 (2015).

[b40] Ramana C. V., Massot M. & Julien C. M. XPS and Raman spectroscopic characterization of LiMn_2_O_4_ spinels. Surf. Interface Anal. 37, 412–416 (2005).

[b41] Ngo D.-T. . Uniform GeO_2_ dispersed in nitrogen-doped porous carbon core-shell architecture: an anode material for lithium ion batteries. J. Mater. Chem. A, 3, 21722–21732 (2015).

[b42] Dokko K., Mohamedi M., Umeda M. & Uchida I. Kinetic Study of Li-Ion Extraction and Insertion at LiMn_2_O_4_ Single Particle Electrodes Using Potential Step and Impedance Methods. J. Electrochem. Soc. 150, A425–A429 (2003).

[b43] Liu Y. . Facile synthesis of nanostructured vanadium oxide as cathode materials for efficient Li-ion batteries. J. Mater. Chem. 22, 24439–24445 (2012).

[b44] Ilango P. R., Prasanna K., Subburaj T., Jo Y. N. & Lee C. W. Facile longitudinal unzipping of carbon nanotubes to graphene nanoribbons and their effects on LiMn_2_O_4_ cathodes in rechargeable lithium-ion batteries. Acta Mater. 100, 11–18 (2015).

[b45] Lee W. J. . Nitrogen-doped carbon nanotubes and graphene composite structures for energy and catalytic applications. Chem. Commun. 50, 6818–6830 (2014). 10.1039/c4cc00146j 24710592

[b46] Czerw R. . Identification of Electron Donor States in N-Doped Carbon Nanotubes. Nano Lett. 1, 457–460 (2001).

[b47] Wang X. . Atomistic Origins of High Rate Capability and Capacity of N-Doped Graphene for Lithium Storage. Nano Lett. 14, 1164–1171 (2014). 24479759 10.1021/nl4038592

[b48] Wu X., Chen S., Ma M. & Liu J. Synthesis of Co-coated lithium manganese oxide and its characterization as cathode for lithium ion battery. Ionics 17, 35–39 (2011).

[b49] Gao X. . Combustion-derived nanocrystalline LiMn_2_O_4_ as a promising cathode material for lithium-ion batteries. J. Power Sources 275, 38–44 (2015).

[b50] L.-H. Hu, B., Wu F.-Y., Lin C.-T., Khlobystov A. N. & Li L.-J. Graphene-modified LiFePO_4_ cathode for lithium ion battery beyond theoretical capacity. Nat. Commun. 4, 1687 (2013). 23575691 10.1038/ncomms2705

[b51] Lee W. J. . Depth profile studies on nickel rich cathode material surfaces after cycling with an electrolyte containing vinylene carbonate at elevated temperature. Phys. Chem. Chem. Phys. 16, 17062–17071 (2014). 25005044 10.1039/c4cp02075h

[b52] Sahan H., Göktepe H. & Patat S. A Novel Method to Improve the Electrochemical Performance of LiMn_2_O_4_ Cathode Active Material by CaCO_3_ Surface Coating. J. Mater. Sci. Technol. 27(5), 415–420 (2011).

[b53] Myung S.-T. . Improvement of cycling performance of Li_1.1_Mn_1.9_O_4_ at 60 °C by NiO addition for Li-ion secondary batteries. Electrochim. Acta 51, 5912–5919 (2006).

[b54] Myung S.-T. . Role of Alumina Coating on Li-Ni-Co-Mn-O Particles as Positive Electrode Material for Lithium-Ion Batteries. Chem. Mater. 17, 3695–3704 (2005).

[b55] Jin G. . Synthesis of single-crystalline octahedral LiMn_2_O_4_ as high performance cathode for Li-ion battery. Electrochim. Acta 150, 1–7 (2014).

[b56] Bai Z., Fan N., Ju Z., Sun C. & Qian Y. LiMn_2_O_4_ nanorods synthesized by MnOOH template for lithium-ion batteries with good performance. Mater. Lett. 76, 124–126 (2012).

