# Supplementary material for: Eco-friendly nitrogen-containing carbon encapsulated LiMn2O4 cathodes to enhance the electrochemical properties in rechargeable Li-ion batteries
Source: Sci Rep. 2016 Jul 13;6:29826. doi: 10.1038/srep29826 (PMC4942828; doi:10.1038/srep29826)
Supplement: Supplementary Information [file srep29826-s1.pdf]

Supplementary Information

Eco-friendly nitrogen-containing carbon encapsulated $LiMn_2O_4$ cathodes to enhance the electrochemical properties in rechargeable Li-ion batteries

P. Robert Ilango, K. Prasanna, Su Jung Do, Yong Nam Jo, Chang Woo Lee*

Department of Chemical Engineering, College of Engineering, Kyung Hee University, 1732 Deogyeong-daero, Gihung, Yongin, Gyeonggi 17104, South Korea

*Corresponding author tel.: +82-31-201-3825, fax: +82-31-204-8114

E-mail address: cwlee@khu.ac.kr (Chang Woo Lee)

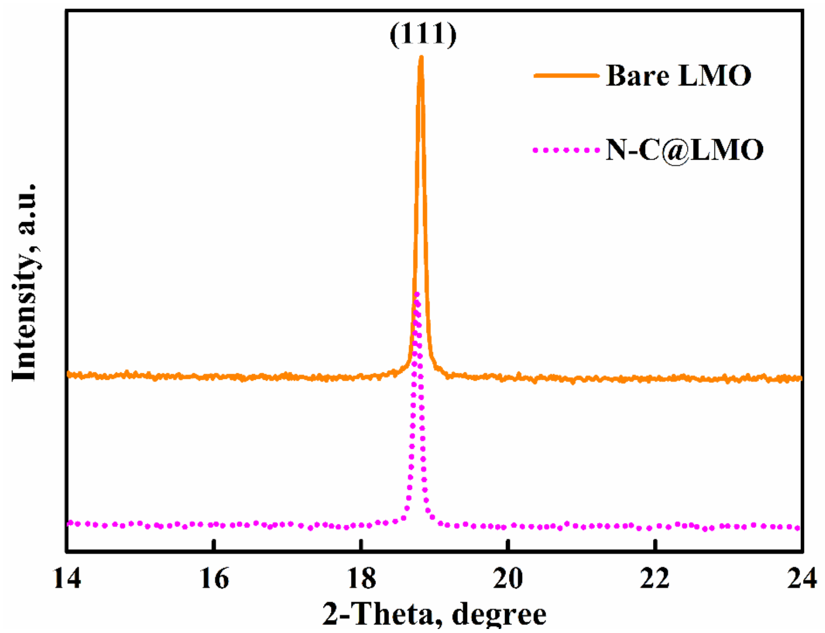

Fig. S1. The magnified view of 111 planes for bare LMO and N-C@LMO samples.

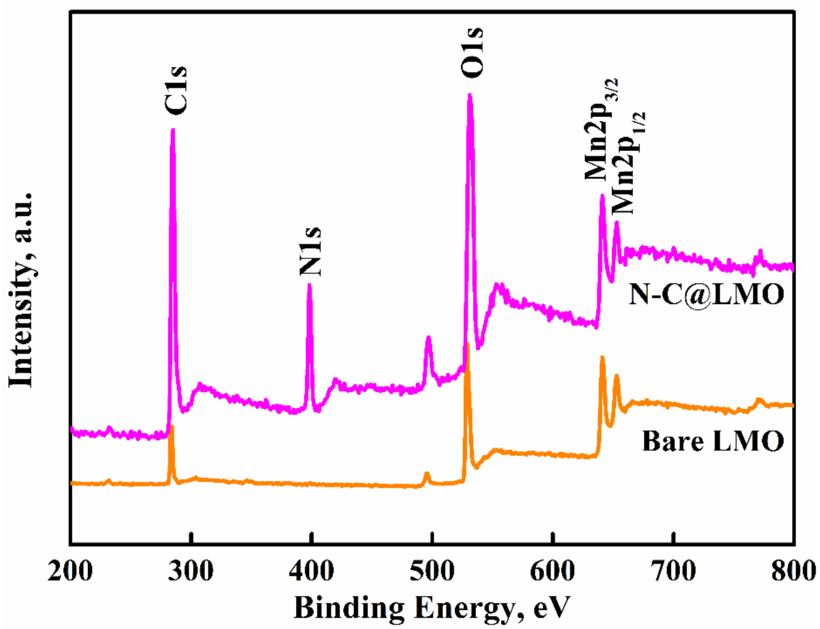

Fig. S2. The XPS survey scan spectra for bare LMO and N-C@LMO samples.

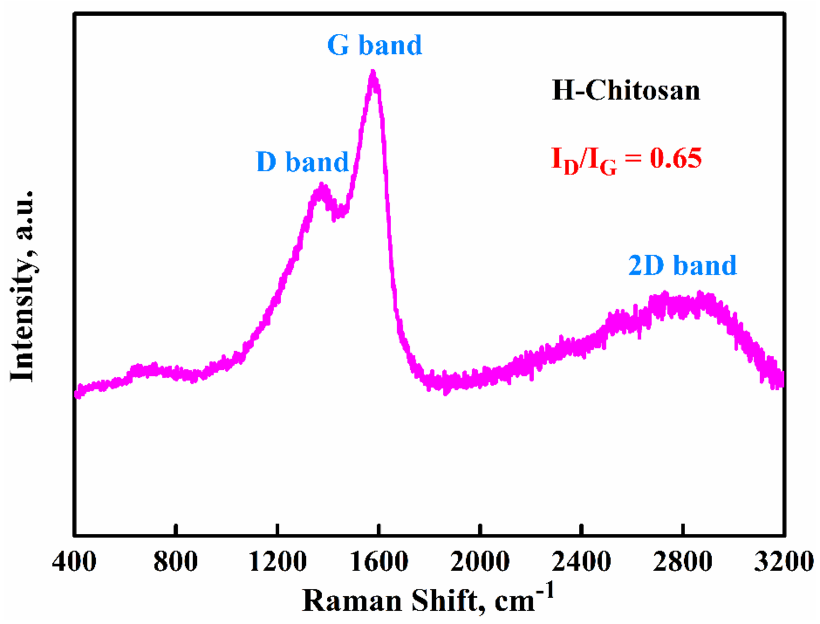

Fig. S3. The HR-Raman spectrum for H-Chitosan.
